# Could Acting Training Improve Social Cognition and Emotional Control?

**DOI:** 10.3389/fnhum.2020.00348

**Published:** 2020-09-23

**Authors:** Brennan McDonald, Thalia R. Goldstein, Philipp Kanske

**Affiliations:** ^1^Clinical Psychology and Behavioral Neuroscience, Faculty of Psychology, Technische Universität Dresden, Dresden, Germany; ^2^Department of Psychology, George Mason University, Fairfax, VA, United States; ^3^Max Planck Institute for Human Cognitive and Brain Sciences, Leipzig, Germany

**Keywords:** theory of mind, empathy, mentalizing, emotion regulation, perspective-taking

Acting is fascinating from psychological and neuroscientific perspectives, as it involves an individual creating an endogenously generated, accurate physical and verbal performance of another's emotional and cognitive states. However, despite the popularity of acting, the practice has received limited interest from cognitive neuroscience (Goldstein and Bloom, 2011, although see Brown et al., 2019), while other art forms have raised much greater attention, including music (e.g., Koelsch, 2014), visual art (e.g., Bolwerk et al., 2014), literature (e.g., Jacobs, 2015), poetry (e.g., Zeman et al., 2013), and dance (e.g., Karpati et al., 2017). Nevertheless, acting requires a range of social, cognitive and affective skills of concern to neuroscience, including memory, verbal ability, emotional control and social cognitive processes like empathy and Theory of Mind (ToM; Noice and Noice, 2006; Goldstein and Winner, 2012; Winner et al., 2013).

Two questions are of particular interest: (i) What are the neural mechanisms that allow actors to produce realistic performances of characters other than themselves? (ii) What long-term impact does acting training have on (social) neurocognition? Following Goldstein and Winner (2012), we explore how neuroscientific research into ToM, empathy, and emotional processing, is beginning to illuminate how actors manifest characters. Additionally, we propose that engagement with acting may in turn improve social competencies by inducing changes in the neural networks underlying social cognition.

## Approaches to Acting

Debate over which techniques allow actors to produce realistic performances has a long history, with numerous schools theorizing and prescribing methods for acting practice (for review see Noice and Noice, 2013; Gallagher and Gallagher, 2019). These include debates surrounding whether an actor should (i) understand/elaborate on a character's mental life, draw on personal experience and replicate the emotions of the character during performance (Stanislavski, 2013), (ii) remain detached, perfecting and portraying the character's outward behaviors (Diderot, 1957), or (iii) actively embody the character, truthfully experiencing emotions within the imaginary world of the performance (Meisner and Longwell, 1987). Importantly, the approach an actor takes to create and perform a character will likely influence the cognitive and affective processes involved. As our intent is to provide a brief commentary on neuroscience's role in understanding acting, we avoid committing to any specific approach, instead understanding acting in the broadest sense as the art or practice of representing a character for the purpose of performance (Merriam-Webster., 2018). However, it is worth keeping in mind that many approaches to acting exist and that future neuroscientific research on acting must take this into account.

## Theory of Mind and Empathy

The ability to represent others' mental states, referred to as ToM or cognitive empathy, plays a critical role in understanding and navigating social situations (Frith and Frith, 2006). ToM represents a socio-cognitive phenomenon, involving abstract, propositional knowledge about another's mental state (Happe et al., 2017; Kanske, 2018; Preckel et al., 2018). Depending on the actor's approach to character performance, reflection into the character's mental life may be important part of the character creation process, involving exploration of the history, motivations, beliefs and values of the person to be portrayed, often going well beyond the information contained within the script (Noice and Noice, 2013). In other words, for many actors, creating a character involves the complex application of ToM. Brain regions involved in ToM include superior temporal sulcus, temporal poles, medial prefrontal cortex, temporoparietal junction, and posterior cingulate/precuneus (Schurz et al., 2014). Additionally, ventromedial prefrontal cortex and temporoparietal junction are implicated in self/other processing and judgments (Denny et al., 2012) and it has been suggested that overcoming self–other interference and ToM are deeply integrated processes (Qureshi et al., 2020). Moreover, simulating others has been shown to influence self-knowledge, with trait and memory measures becoming similar to a simulated other after adopting their perspective (Meyer et al., 2019). In relation to acting, a recent neuroimaging study demonstrated that when trained actors answered questions from the first-person fictional perspective of a character, in contrast to their own perspective, dorsomedial prefrontal cortex/superior frontal gyrus and ventromedial prefrontal cortex are deactivated, suggesting acting may involve the suppression of self-processing (Brown et al., 2019). Concurrently, increased activation was found in the precuneus, a region belonging to the brain's dorsal attentional network, involved in episodic retrieval, attentional orienting, and visual imagery (Fletcher et al., 1995; Cavanna and Trimble, 2006; Spreng et al., 2010). Interestingly, a similar pattern of deactivation in prefrontal regions was observed when the actors were asked to respond to questions in their own perspective while adopting a British accent compared to no accent; however, no precuneus activation was observed for this contrast. Brown and colleagues interpret this finding by suggesting that the pretense of adopting an (unspecified) other's attributes may require suppression of the self, while acting out a specified character could require an additional dispersion of self-related attentional resources. Interestingly, they relate this proposed dispersion to the idea of *split* or *duel consciousness*, an important concept in acting theory (Diderot, 1957; Stanislavski, 2013), describing the constant shifting of an actor's attention between the conscious awareness of the self during the performance and the perspective of the character existing in the world of the play. Brown et al. thus argue that precuneus activation may reflect the attentional maintenance of the actor's identity as a conscious self while adopting the fictional character's perspective.

In contrast to ToM, empathy (also referred to as emotional or affective empathy) has been defined as sharing or mirroring the feelings of another, while being aware that the emotion originates from another person (De Vignemont and Singer, 2006). Neurally, empathy involves activation of the same brain networks during the shared emotion as would be active during a first-hand experience (Gallese, 2003; Wicker et al., 2003; Singer and Lamm, 2009). For example, the experience of pain and witnessing another experiencing pain results in the activation of a core network consisting of the anterior insula and anterior cingulate cortex (Singer et al., 2004; Jackson et al., 2005; Corradi-Dell'Acqua et al., 2016). Importantly, neuroimaging evidence has demonstrated that the underlying neural mechanisms involved in empathy and ToM are distinct and separately contribute to social competencies (Blair, 1995, 2005; Kanske et al., 2015, 2016). The role of empathy in acting practice has long been an important point of theoretical discussion (for review see Gallagher and Gallagher, 2019). Moreover, empirical work suggests that acting training may improve behavioral measures of empathy (Goldstein and Winner, 2012). However, the means by which the neural networks underlying empathy are recruited/altered during acting remain unknown. Given the perceived importance of empathy in acting practice, a neuroimaging approach may offer novel insight into the role socio-affective phenomena play during dramatic performance.

## Emotion Regulation and Generation

During performance an actor must portray a character, including facial expressions, vocal inflection, movement, and body language. In addition, the actor often has to reflect and respond to other performers on stage. Under these conditions, an actor must not only empathize with and take the perspective of the character, but also regulate and spontaneously generate appropriate emotional states. The process of emotion regulation involves altering ongoing emotional states by employing regulatory cognitive processes; including producing changes in attention and applying cognitive control strategies (Gross, 1998, 2002; Ochsner and Gross, 2005; Kanske et al., 2011). Importantly, and depending on circumstance, there are more or less adaptive/dysfunctional ways of regulating emotions (e.g., acceptance or suppression of an emotional experience) and successful emotion regulation is linked to a range of psychological, social and physical health outcomes (Gross, 2002, 2008). For example, Gross and Levenson (1993) demonstrated that the sympathetic nervous system activity of individuals instructed not to show emotion through facial expressions when presented with disgusting stimuli was higher than in individuals allowed to display disgust through their face, thus suggesting that acceptance of negative emotions may be more beneficial than emotional suppression. Additionally, studies have begun to demonstrate that affective responses may be modified by consciously taking the perspective of other people (i.e., ToM; Gilead et al., 2016; McDonald et al., 2020). For example, Gilead and colleagues showed that activity in the medial prefrontal cortex and amygdala of participants taking the perspective of either a sensitive/squeamish or tough/resilient individual differentially simulated the expected negative affective state of the target. This finding suggests that taking another's perspective impacts our own ability to regulate and experience emotions. Given that actors must regulate their emotions when rehearsing and performing a character, we propose that they may be more proficient in adopting and utilizing emotion regulation strategies. In a similar vein, Goldstein et al. (2009) has suggested that actors are likely to be more accepting of their emotions and less likely engage in emotional suppression. Goldstein et al. (2013) found that a year of acting practice decreased the use of emotional suppression in children aged 7–10, while adolescents majoring in acting at high school (compared to other art majors e.g., music) used less suppression. Additionally, 4–5-years olds randomly assigned to an 8-week drama condition (compared to block building or reading) showed increased emotional control (i.e., inhibition of affective responses to observed or discussed distress; Goldstein and Lerner, 2018).

Complementary to the process of emotion regulation is the process of endogenous emotion generation, which involves emotions being experienced by an individual as a result of internal cognitive and affective processes, often in the absence of external stimuli. In the context of acting, emotion generation is usually a very deliberate, voluntary process occurring in the specific contexts of a performance, often with other actors present (Noice and Noice, 2013). Engen et al. (2017) examined participants endogenously generating emotions during functional neuroimaging and found that the brain's salience network (involved in detecting and filtering task-relevant stimuli; Seeley et al., 2007), including the anterior insula and dorsomedial prefrontal cortex, as well as basal ganglia and midbrain structures, were implicated in initial affect generation. In contrast, default mode and frontoparietal control networks (implicated primarily in non-task related, resting states and cognitive control processes, respectively; Spreng et al., 2010) were involved in elevated affect even after active generation had ceased, with the emotional states only being deactivated when suppressed by the participants. These findings thus demonstrate that people are in principle capable of spontaneously generating emotional states, as well as manipulating and regulating their emotions by adopting another's perspective.

Importantly, however, actors vary in the techniques used to portray the emotions of a character. If an actor is inclined to actively embody the character's emotional state this will likely require different emotion regulation/generation strategies and neural processes (e.g., De Gelder, 2006; Niedenthal, 2007; Nummenmaa et al., 2014) than an actor that uses simulation to portray the emotional behaviors (e.g., Decety and Grèzes, 2006). Thus, any comprehensive understanding of emotion regulation and generation in actors must take into account the actor's approach to performing emotions. Based on this, a goal of future research should be to examine the neural underpinnings and overall impact of emotional processing across the acting experience. Finally, acting could also present a means to examine the complex interplay between bottom-up empathic mirroring of emotions and top-down emotional regulation, which partially share underlying neural circuitry (Singer and Lamm, 2009; Ochsner et al., 2012) and may be deeply interconnected processes.

## The Impact of Acting

A growing body of evidence shows that socio-affective and socio-cognitive brain networks are principally plastic, with interventions such as mental training practices (e.g., affect- or metacognition-focused meditation) inducing changes in cortical morphology (Valk et al., 2017; Trautwein et al., 2020). With respect to acting, Schellenberg (2004) showed that 6-year-old children assigned with 6 weeks of acting training had improved behavioral ToM measures compared to children assigned to 6 weeks of music practice. Similarly, Goldstein et al. (2009) showed that adolescent and adult actors have above average skill in ToM tasks, but not above average levels of empathy. Somewhat contrasting with this result, Goldstein and Winner (2012) followed children and adolescents receiving 1 year of either acting or other arts training (i.e., visual arts, music) and assessed empathy and ToM before and after training. In both groups, those receiving acting (but not other arts) training showed significant gains in empathy and ToM. Additionally, Nettle (2006) demonstrated that actors score slightly higher levels of empathy than non-actor controls. These findings suggest that both ToM and empathy may be amenable to improvement through acting training, however, a goal of further research should be to tease apart the conditions (e.g., age, acting experience, acting techniques, personality measures) under which such improvements occur.

Based on these initial findings, we propose that actors, by repeatedly engaging the first-person fictional perspective required to produce a character, may induce changes to the cortical networks underlying social cognition. Such changes could occur as the result of Hebbian learning mechanisms which involve the strengthening of neural network functional and effective connectivity due to frequent network engagement (Keysers and Gazzola, 2006). Moreover, specific strengthening of these social networks may crucially contribute to improvements in an actor's ability to portray a particular character. Finally, by engaging in different characters on a regular basis, an actor is exposing the networks underlying ToM, empathy and emotional processing to a broad variety of novel social stimuli. It is via repeated exposure that, we contend, generalized improvements in social cognitive abilities arise from prolonged engagement with acting.

Taken together, these initial behavioral studies suggest that engagement in acting training may indeed improve aspects of social cognition. Given that many mental disorders involve impairments in social cognition (Gallagher and Varga, 2015) and that social abilities are important in both educational and business settings (Blakemore, 2010; Hülsheger and Schewe, 2011), we suggest that future research into acting's psychological impact may offer new avenues to understand and improve social skills. For example, there is a burgeoning literature exploring theatrical techniques as a possible intervention for autism spectrum disorders (Corbett et al., 2011, 2019; Gabriel et al., 2016), while a recent study showed 6 weeks of improve theater training produced increased creativity and psychological well-being in participants (Schwenke et al., 2020). Finally, we promote a broader discussion between the fields of acting theory/pedagogy and cognitive neuroscience, as we believe exchange between these disciplines will provide both a deeper understanding of the actor's craft as well as motivate novel insights into the neural networks underlying ToM, empathy and emotional processing.

## Author Contributions

BM, TG, and PK contributed to the conception and writing of this review. BM prepared the draft for submission. All authors contributed to the article and approved the submitted version.

## Conflict of Interest

The authors declare that the research was conducted in the absence of any commercial or financial relationships that could be construed as a potential conflict of interest.
